# Association between triglyceride-glucose and triglyceride glucose body mass index with risk of prediabetes: a multicenter Chinese medical examination cohort study

**DOI:** 10.3389/fendo.2025.1668021

**Published:** 2025-10-10

**Authors:** Chengya Feng, Xinxing Wang, Yanni Luo

**Affiliations:** ^1^ Department of Clinical Laboratory, Deyang Jingyang Maternal and Child Health Hospital, Deyang, Sichuan, China; ^2^ Department of Clinical Laboratory, Chengdu BOE Hospital, Chengdu, Sichuan, China; ^3^ Department of Clinical Laboratory, The First People’s Hospital of Nanning, Nanning, China

**Keywords:** triglyceride-glucose index, triglyceride-glucose–body mass index, prediabetes, Chinese adults, cohort study

## Abstract

**Background and aims:**

Although triglyceride-glucose (TyG) indices reliably identify insulin resistance and diabetes, their link to prediabetes risk is understudied. We analyze the associations of TyG and TyG-body mass index (TyG-BMI) with prediabetes risk in a Chinese population.

**Methods:**

This retrospective cohort study used data from 11 urban physical-examination centers of the Rich Healthcare Group. A total of 161606 Chinese adults free of diabetes at baseline were included. Participants were categorized by quartiles of the TyG index and the TyG-BMI. Cox proportional hazards regression and Kaplan–Meier analyses were used to estimate the associations between these exposures and incident prediabetes. Restricted cubic splines with piecewise Cox models were applied to explore potential non-linear relationships and identify inflection points. Subgroup and sensitivity analyses were performed to assess the robustness of the findings.

**Results:**

Over a median follow-up of 3.0 years, 18339 participants (11.3%) developed incident prediabetes. After multivariable adjustment, both TyG and TyG-BMI were positively associated with prediabetes risk. Restricted cubic splines revealed non-linear relationships (*P* < 0.001) with distinct threshold effects. The associations were stronger among adults younger than 45 years and among women, with significant additive interactions observed. Kaplan–Meier curves showed the highest cumulative incidence of prediabetes in the top quartiles of both TyG and TyG-BMI.

**Conclusions:**

In this Chinese cohort, TyG and TyG-BMI showed non-linear associations with incident prediabetes, with steeper risk increases among women and adults younger than 45 years. These indices may identify high-risk insulin resistance states preceding dysglycemia.

## Introduction

Diabetes mellitus is a prevalent chronic metabolic disease worldwide and is frequently accompanied by serious complications (1). Its onset is typically preceded by a prodromal state—prediabetes—defined by elevated but nondiabetic blood glucose levels. Even at this stage, the risk of both micro- and macrovascular complications is markedly increased (2). Globally, more than 400 million adults currently have prediabetes, a figure projected to exceed 600 million by 2045 (3). In China, the age-standardized prevalence among adults is 35.7%, surpassing that of most other chronic disorders (4). Critically, intensive lifestyle or pharmacologic interventions can restore normoglycemia in a substantial proportion of individuals within a relatively short period, and the benefits accrue over time. In the Diabetes Prevention Program Outcomes Study, participants who reverted to normal glucose regulation—even once—experienced a ≥50% reduction in the subsequent risk of type 2 diabetes (T2D) (5, 6) and parallel declines in cardiovascular events, microvascular damage, nephropathy, and retinopathy (7). Given the substantial public health burden imposed by diabetes and its prequel state, a deeper understanding of the determinants of prediabetes is essential for the early identification of high-risk individuals and the timely deployment of effective preventive strategies.

Insulin resistance (IR), characterized by chronic compensatory hyperinsulinemia, is the fundamental pathophysiological driver of prediabetes and an independent risk factor for multisystem complications including cardiovascular disease, cellular senescence, and neurodegeneration, often preceding dysglycemia by years (8, 9). Direct quantification with the euglycemic-hyperinsulinemic clamp or intravenous glucose tolerance test is prohibitively expensive and technically demanding, precluding large-scale screening (10, 11). The triglyceride–glucose (TyG) index—calculated from fasting triglycerides (TG) and fasting plasma glucose (FPG)—has emerged as a practical and cost-effective surrogate marker of IR (12, 13)and has consistently demonstrated robust associations with incident T2D and cardiovascular events across diverse populations. More recently, obesity-adjusted derivatives such as the TyG-body mass index (TyG-BMI) have been developed to integrate IR with visceral adiposity, yielding superior metabolic risk prediction (14). Nevertheless, prior investigations have been limited by single-center designs and modest sample sizes, and large-scale studies systematically evaluating the predictive value of the TyG index and its obesity-adjusted variants for prediabetes in the Chinese population are lacking.

Although prior studies have linked the TyG index and its derivatives to incident diabetes mellitus (15, 16), their longitudinal value for identifying the earliest dysglycemic transition—prediabetes—remains undefined. Using a multicenter, retrospective Chinese cohort, we will quantify the temporal associations and predictive performance of TyG and TyG-BMI for future prediabetes onset, aiming to deliver an evidence-based tool for early identification of high-risk IR states.

## Methods

### Study design and data source

**Figure 1:**
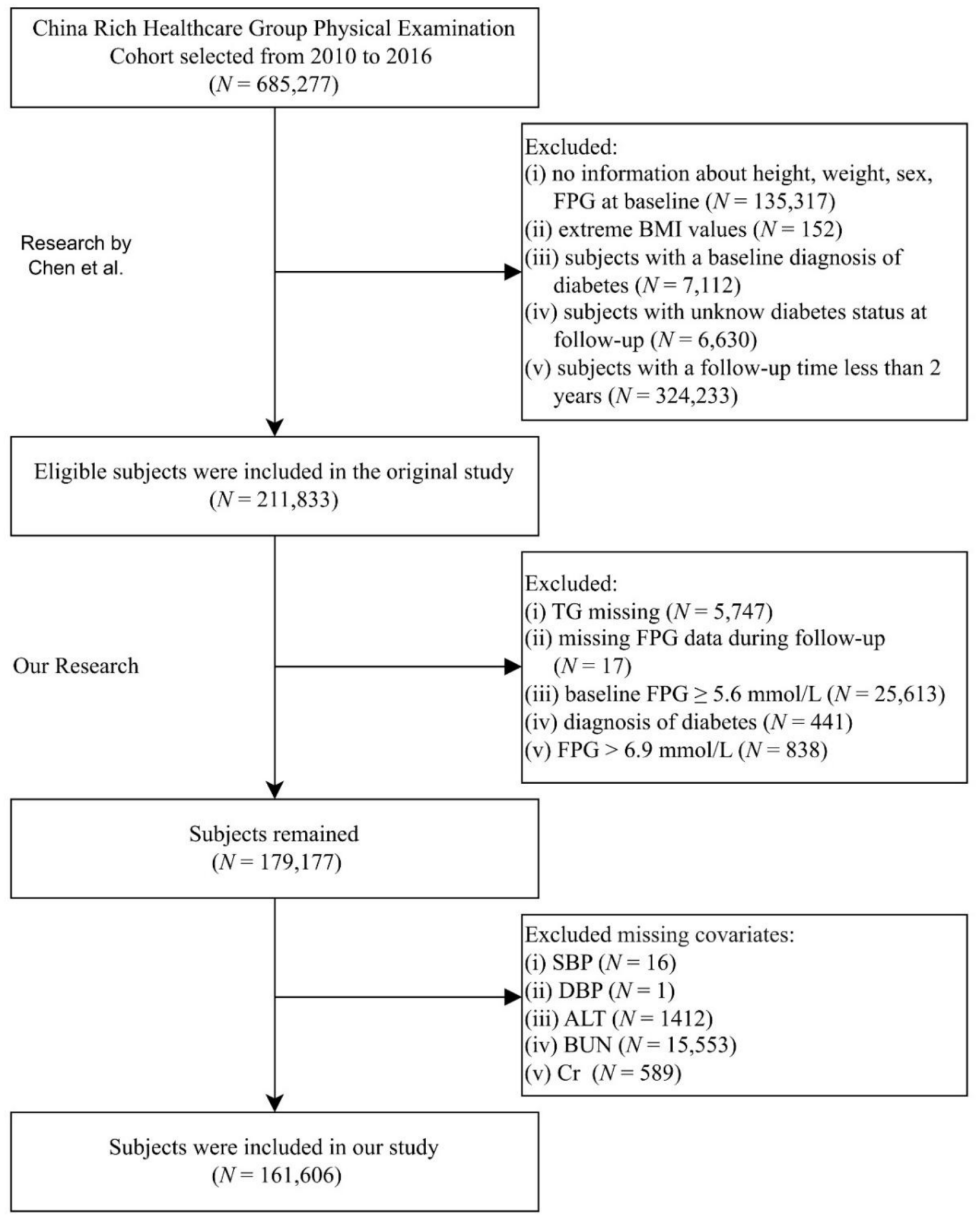
Participant inclusion flowchart. FPG, fasting plasma glucose; BMI, body mass index; TG, triglyceride; SBP, systolic blood pressure; DBP, diastolic blood pressure; ALT, alanine aminotransferase; BUN, blood urea nitrogen; Cr, creatinine.

De-identified records were obtained from the Dryad repository (https://datadryad.org/dataset/doi:10.5061/dryad.ft8750v). Under Dryad’s reuse policy, secondary analyses are permitted; the parent study received institutional review board approval, precluding additional ethical review. The repository contains 685,227 adults (age ≥20 years) who underwent routine health examinations at Rich Healthcare Group between 2010 and 2016 (17). Li et al. previously cleaned the data by excluding individuals with incomplete covariates, extreme body mass index, <2 years of follow-up, prevalent diabetes, or indeterminate glycemic status, yielding a cohort of 211,833 participants. For the present study, we applied the American Diabetes Association prediabetes criteria to this curated dataset and sequentially excluded participants with (1) missing TG (n = 5,747), (2) missing follow-up glucose (n = 17), (3) baseline FPG ≥5.6 mmol/L (n = 25,613), (4) self-reported physician-diagnosed diabetes or follow-up FPG >6.9 mmol/L (n = 1,279), and (5) incomplete covariates (n = 17,571). After exclusions, 161,606 individuals remained for analysis. **Figure 1** depicts the selection flow; **Supplementary Table 1** lists missing-data details.

### Clinical index measurement

Participants completed a uniform questionnaire that recorded family history of diabetes and demographic variables (sex, age). Height and weight were measured in a temperature-controlled room with calibrated stadiometers and digital scales, respectively; body mass index (BMI) was calculated as weight in kilograms divided by the square of height in meters. Blood pressure was obtained in a quiet environment after a 5-minute rest using a standard mercury sphygmomanometer. Following a 10-hour overnight fast, trained phlebotomists collected venous blood. All analytes were processed on a Beckman Coulter AU5800 automated analyzer. FPG was determined by the glucose oxidase method. TG, alanine aminotransferase (ALT), creatinine (Cr), and blood urea nitrogen (BUN) were quantified using optical turbid metric assays. The TyG index was computed as ln [FPG (mg/dL) × TG (mg/dL)/2] (18), and the TyG-BMI index as TyG × BMI (19).

### Diagnosis of prediabetes

According to the 2018 American Diabetes Association Standards of Medical Care in Diabetes, participants were defined as having prediabetes if they developed a FPG level between 5.6 mmol/L and 6.9 mmol/L during follow-up and did not progress to meet the diagnostic criteria for diabetes mellitus (20).

### Statistical analysis

All statistical analyses were conducted using R version 4.2.2 (R Foundation) and Free Statistics version 2.2.0. The statistical significance was set at *P* < 0.05 (bilateral).

Normally distributed continuous variables among groups were compared with one-way ANOVA and are presented as mean ± Standard Deviation (SD); non-normally distributed variables were analyzed with the Kruskal–Wallis test and are reported as median (interquartile range). Categorical variables were compared using the χ² test and are expressed as percentages.

Because missing data were minimal (range 0% to 8.9%), no imputation was performed. Multivariable Cox proportional hazards models were constructed to estimate hazard ratios (HRs) and 95% CIs for the Diagnosis of prediabetes associations of TyG and TyG-BMI with incident prediabetes. Model 1 was unadjusted. Model 2 adjusted for age, sex, and family history of diabetes. Model 3 added systolic and diastolic blood pressure. Model 4 further included ALT, BUN, and Cr.

To assess potential non-linear associations, restricted cubic splines for TyG and TyG-BMI were fitted within Model 4; departure from linearity was evaluated by likelihood ratio tests comparing the spline model with a linear term. Where non-linearity was detected, piecewise Cox regression was used to estimate threshold effects. Effect modification by age (< 45 vs ≥ 45 years), sex, and family history of diabetes was examined by including cross-product terms in Cox models; interaction significance was assessed with likelihood ratio tests. Additive interactions were quantified using the relative excess risk due to interaction (RERI), attributable proportion (AP), and synergy index (SI), with 95% CIs derived via the delta method. Kaplan–Meier curves illustrate cumulative prediabetes incidence across TyG and TyG-BMI categories.

To ensure the robustness of our findings, we conducted five complementary sensitivity analyses: (1) multiple imputation by chained equations was applied to all covariates with missing values; (2) participants with a family history of diabetes at baseline were excluded and the multivariable Cox proportional-hazards model was refitted; (3) individuals with abnormal blood pressure at baseline were further removed and the analysis repeated; (4) TyG and TyG-BMI were recoded from continuous to categorical variables using quintiles to re-examine dose–response relationships; and (5) both indices were winsorized at the 0.5th and 99.5th percentiles to minimize the influence of extreme values.

## Results

### Baseline characteristics of subjects

This study included 161,606 adults (mean [SD] age, 41.2 [12.2] years), among whom 86393 (53.5%) were male. The mean (SD) TyG index was 8.3 (0.6), and the mean (SD) TyG-BMI was 192.2 (35.7). During follow-up, 18,339 incident cases of prediabetes were recorded, accounting for 11.3% of the cohort. **Table 1** stratifies participants by TyG index quartiles. Higher TyG quartiles were associated with progressively increased levels of systolic and diastolic blood pressure, BMI, FPG, TG, ALT, BUN, and Cr. The proportion of participants reporting a family history of diabetes did not differ significantly across TyG quartiles (*P* = 0.162).

**Table 1:** Baseline characteristics of the study participants stratified by TYG quartile.

Variables	Total	Q1 (<7.90)	Q2 (7.90-8.27)	Q3 (8.27-8.69)	Q4 (≥8.69)	*P*-value
Participants, *N*	161606	40065	40423	40513	40605	
Demographic						
Age, y	41.2 ± 12.2	37.1 ± 9.7	39.9 ± 11.6	42.6 ± 12.8	45.1 ± 12.8	< 0.001
Sex, n (%)						< 0.001
Male	86393 (53.5)	12874 (32.1)	18798 (46.5)	24368 (60.1)	30353 (74.8)	
Female	75213 (46.5)	27191 (67.9)	21625 (53.5)	16145 (39.9)	10252 (25.2)	
Family history of diabetes, n (%)						0.162
No	158344 (98.0)	39309 (98.1)	39586 (97.9)	39693 (98.0)	39756 (97.9)	
Yes	3262 ( 2.0)	756 (1.9)	837 (2.1)	820 (2.0)	849 (2.1)	
Health status						
SBP, mmHg	117.9 ± 15.9	112.0 ± 13.9	115.7 ± 14.9	119.6 ± 15.7	124.3 ± 16.1	< 0.001
DBP mmHg	73.6 ± 10.6	69.8 ± 9.5	72.0 ± 9.9	74.5 ± 10.4	77.9 ± 10.8	< 0.001
Anthropometric measures						
Height, cm	166.5 ± 8.3	164.5 ± 7.8	165.8 ± 8.3	167.0 ± 8.4	168.5 ± 8.2	< 0.001
Weight, kg	64.1 ± 12.0	57.6 ± 9.4	61.4 ± 10.7	65.7 ± 11.5	71.6 ± 11.7	< 0.001
BMI, kg/m^2^	23.0 ± 3.3	21.2 ± 2.6	22.2 ± 2.9	23.5 ± 3.1	25.1 ± 3.1	< 0.001
FPG, mmol/L	4.8 ± 0.5	4.6 ± 0.5	4.7 ± 0.5	4.8 ± 0.4	4.9 ± 0.4	< 0.001
TG, mmol/L	1.0 (0.7, 1.5)	0.6 (0.5, 0.7)	0.9 (0.8, 0.9)	1.2 (1.1, 1.4)	2.1 (1.7, 2.6)	< 0.001
ALT, U/L	17.6 (12.6, 26.7)	13.7 (10.8, 18.5)	15.6 (11.8, 22.2)	19.0 (13.7, 27.5)	25.4 (17.7, 38.3)	< 0.001
BUN, mmol/L	4.6 ± 1.2	4.5 ± 1.2	4.5 ± 1.2	4.6 ± 1.2	4.7 ± 1.1	< 0.001
Cr, umol/L	69.7 ± 15.6	64.5 ± 13.9	68.0 ± 15.7	71.3 ± 15.8	74.9 ± 15.1	< 0.001
TyG	8.3 ± 0.6	7.6 ± 0.2	8.1 ± 0.1	8.5 ± 0.1	9.1 ± 0.3	< 0.001
TyG-BMI	192.2 ± 35.7	161.5 ± 20.7	179.8 ± 23.8	198.6 ± 26.7	228.4 ± 30.7	< 0.001

Data are presented as mean (SD), (IQR) or number (%), as appropriate.

BMI, body mass index; SBP, systolic blood pressure; DBP, diastolic blood pressure; FPG, fasting plasma glucose; TC, total cholesterol; TG; triglyceride; ALT, alanine aminotransferase; BUN, blood urea nitrogen; Cr; creatinine; TyG, triglyceride-glucose index, TyG-BMI, TyG with body mass index.

### Association between TyG index, TyG-BMI, and risk of prediabetes

Univariable analyses revealed significant associations of age, sex, systolic blood pressure, diastolic blood pressure, alanine aminotransferase, blood urea nitrogen, and creatinine with incident prediabetes (**Supplementary Table 2**).

After multivariable adjustment, both TyG and TyG-BMI remained independently associated with incident prediabetes (**Table 2**). Each 1-unit increase in TyG conferred a 68% higher risk (HR, 1.68; 95% CI, 1.63–1.72; *P* < 0.001). Participants in the highest TyG quartile had a 139% greater risk than those in the lowest quartile (*P* < 0.001); the corresponding excess risk for the highest TyG-BMI quartile was 146% (*P* < 0.001).

**Table 2:** Association of TyG and TyG-BMI with the risk of prediabetes.

Categories	Model 1		Model 2		Model 3		Model 4	
	HR (95% CI)	*P* value	HR (95% CI)	*P* value	HR (95% CI)	*P* value	HR (95% CI)	*P* value
TyG	2.12 (2.08~2.17)	<0.001	1.80 (1.76~1.85)	<0.001	1.70 (1.66~1.74)	<0.001	1.68 (1.63~1.72)	<0.001
TyG quartile								
Q1	Reference		Reference		Reference		Reference	
Q2	1.60 (1.52~1.68)	<0.001	1.41 (1.34~1.49)	<0.001	1.37 (1.30~1.44)	<0.001	1.36 (1.29~1.44)	<0.001
Q3	2.38 (2.27~2.50)	<0.001	1.90 (1.80~1.99)	<0.001	1.80 (1.71~1.89)	<0.001	1.78 (1.69~1.87)	<0.001
Q4	3.70 (3.54~3.88)	<0.001	2.69 (2.57~2.83)	<0.001	2.45 (2.33~2.57)	<0.001	2.39 (2.28~2.52)	<0.001
*P* for trend		<0.001		<0.001		<0.001		<0.001
TyG-BMI	1.01 (1.01~1.01)	<0.001	1.01 (1.01~1.01)	<0.001	1.01 (1.01~1.01)	<0.001	1.01 (1.01~1.01)	<0.001
TyG-BMI quartile								
Q1	Reference		Reference		Reference		Reference	
Q2	1.70 (1.61~1.80)	<0.001	1.47 (1.39~1.55)	<0.001	1.42 (1.35~1.50)	<0.001	1.41 (1.34~1.49)	<0.001
Q3	2.65 (2.52~2.79)	<0.001	2.04 (1.93~2.15)	<0.001	1.90 (1.8~2.00)	<0.001	1.87 (1.77~1.97)	<0.001
Q4	3.98 (3.79~4.18)	<0.001	2.89 (2.74~3.05)	<0.001	2.54 (2.41~2.68)	<0.001	2.46 (2.33~2.60)	<0.001
*P* for trend		<0.001		<0.001		<0.001		<0.001

TyG, triglyceride-glucose index, TyG-BMI, TyG with body mass index; HR, hazard ratio; CI, confidence interval.

Model 1: not adjusted.

Model 2: adjusted for age, sex, family history of diabetes.

Model 3: adjusted for model 2, additionally adjusted for systolic blood pressure, diastolic blood pressure.

Model 4: adjusted for model 3, additionally adjusted for alanine aminotransferase, blood urea nitrogen, creatinine.

### Curve fitting and inflection point analysis of TyG-related indices with prediabetes

**Figure 2:**
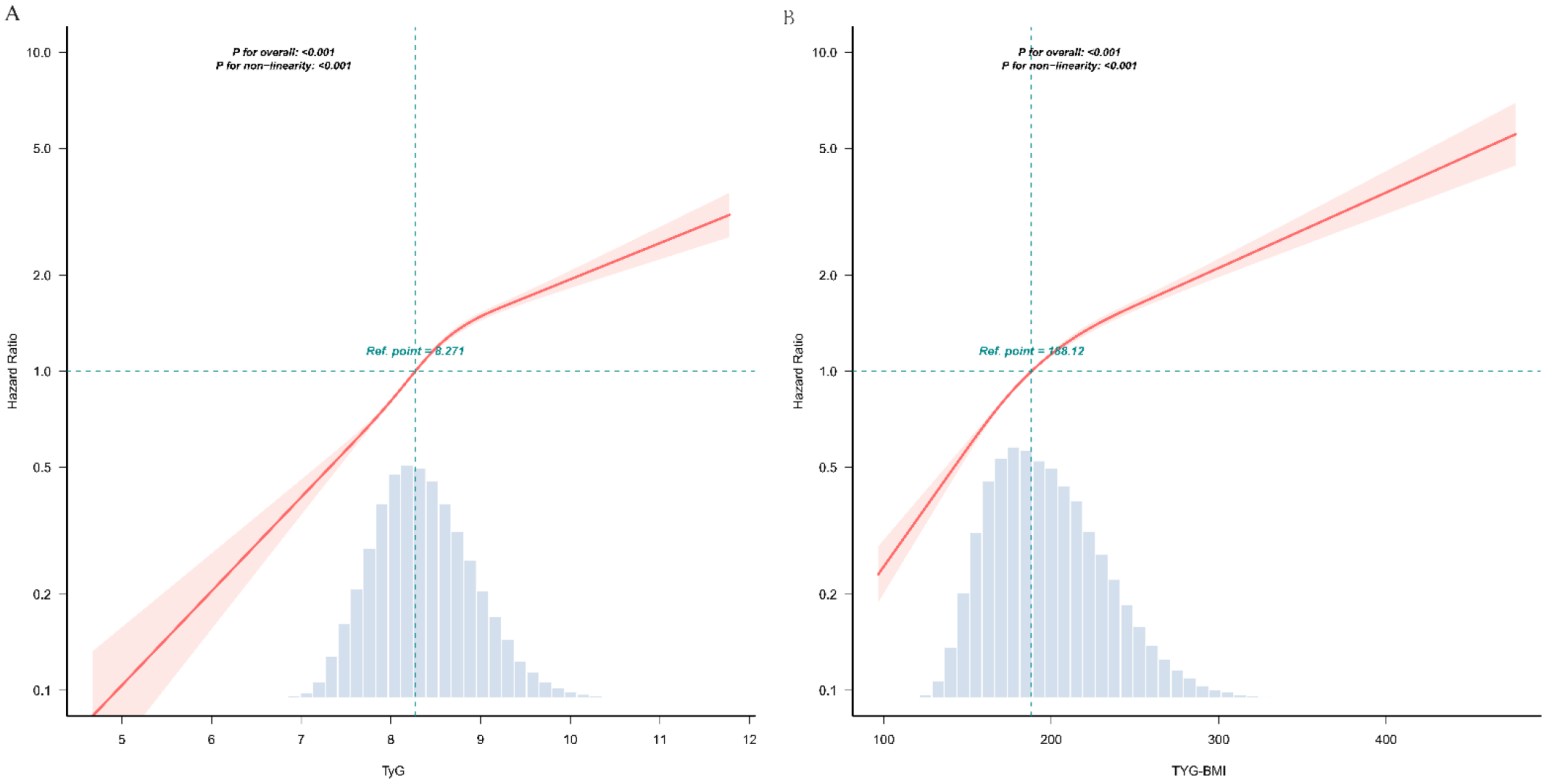
The dose-response associations of TyG **(A)** and TyG-BMI **(B)** with the risk of prediabetes. TyG, triglyceride-glucose index; TyG-BMI, TyG with body mass index This odds ratio plot, constructed with restricted cubic splines, depicts the association between TyG **(A)** and TyG-BMI **(B)** the risk of prediabetes. The knots for the splines are positioned at the 5th, 35th, 65th, and 95th percentiles of TyG and TyG-BMI. The reference point is set at a TyG value of 8.271. or TyG-BMI value of 188.12 respectively. The solid red line indicates the estimated odds ratio, with the red shaded area representing the 95% confidence interval. The dashed line at a hazard ratio of 1 serves as the reference for no effect. Both the overall associations are statistically significant (*P* < 0.001), and the *P*-value for non-linearity are both <0.001. The analysis is adjusted for covariates as per Model 4.

Multivariable-adjusted restricted cubic spline curves (**Figure 2**) revealed non-linear associations of both TyG (**Figure 2A**) and TyG-BMI (**Figure 2B**) with incident prediabetes (*P* for non-linearity < 0.001 for each). Piecewise regression analyses (**Table 3**) identified inflection points of 9 for TyG and 200 for TyG-BMI. Below these thresholds, each unit rise in TyG corresponded to a 93% higher risk (HR, 1.93; 95% CI, 1.85–2.01), whereas above the inflection point the HR attenuated to 1.25 (95% CI, 1.14–1.36). Similarly, TyG-BMI displayed a steeper slope below 200 (HR, 1.012; 95% CI, 1.011–1.014) than above 200 (HR, 1.007; 95% CI, 1.006–1.007); all *P* < 0.001.

**Table 3:** Threshold effect analysis of the relationship of TyG, TyG-BMI with the risk of prediabetes.

Breakpoint	HR (95% CI)	*P* value
TyG		
<9	1.93 (1.8510,2.0130)	< 0.001
≥9	1.246 (1.1400,1.3610)	< 0.001
Likelihood Ratio test		<0.001
TyG-BMI		
<200	1.012 (1.0110,1.0140)	<0.001
≥200	1.0065 (1.0057,1.0073)	<0.001
Likelihood Ratio test		<0.001

TyG, triglyceride-glucose index, TyG-BMI, TyG with body mass index; HR, hazard ratio; CI, confidence interval.

This two-piecewise regression revealed a nonlinear threshold association between TyG, TyG-BMI and prediabetes. Adjusted for age, sex, family history of diabetes, systolic blood pressure, diastolic blood pressure, alanine aminotransferase, blood urea nitrogen, creatinine.

### Subgroup analysis, interaction effects and Kaplan–Meier curves

**Figure 3:**
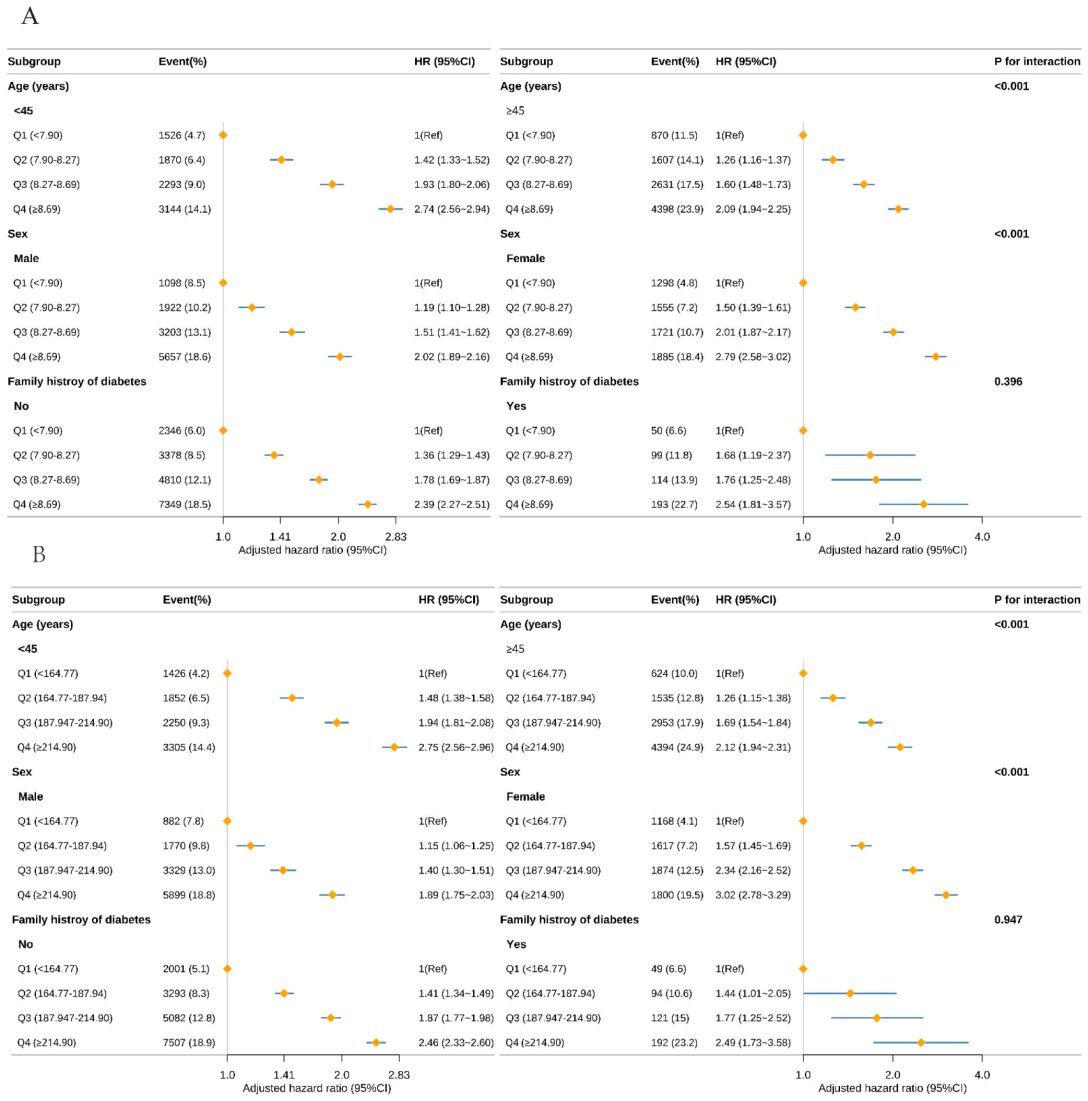
Subgroup analysis of the risk of prediabetes with TyG **(A)**, TyG-BMI **(B)** using multivariable Cox regression model. TyG, triglyceride-glucose index; TyG-BMI, TyG with body mass index Forest plot displaying the adjusted HR and 95% CI for Prediabetes with TyG **(A)**, and TyG-BMI **(B)** across various subgroups. The analysis was conducted using a multivariable Cox regression model. Except for the stratification factor itself, the stratifications were adjusted for all variables adjusting (age, sex, family history of diabetes, systolic blood pressure, diastolic blood pressure, alanine aminotransferase, blood urea nitrogen, creatinine). Subgroup analyses by age, sex, family history of diabetes, and alanine aminotransferase are shown. The diamond represents the overall effect size, and the horizontal lines are the 95% CIs.

To evaluate potential differences in the associations of the TyG index (**Figure 3A**) and TyG-BMI (**Figure 3B**) with incident prediabetes across demographic subgroups, we conducted stratified analyses (**Figure 3**). After stratification by age (<45 years vs ≥45 years), sex (male vs female), and family history of diabetes (no vs yes), significant multiplicative interactions were observed between the TyG index and age (*P* for interaction < 0.001) and between the TyG index and sex (*P* for interaction < 0.001). Specifically, the positive association between the TyG index and prediabetes was stronger among individuals aged <45 years compared with those aged ≥45 years, and stronger among women compared with men. TyG-BMI showed a similar interaction pattern. Further additive interaction analyses (**Supplementary Table 3**) demonstrated that, compared with individuals aged <45 years with a low TyG index (<8.27), those with a high TyG index and aged ≥45 years had a hazard ratio (HR) for prediabetes of 3.02 (95% CI, 2.89–3.15). The relative excess risk due to interaction (RERI) was 0.14 (95% CI, 0.02–0.26), indicating a significant additive interaction. Similarly, significant additive interactions were observed between the TyG index and sex, as well as between TyG-BMI and both age and sex (all RERI >0; 95% CIs excluded 0).

**Figure 4:**
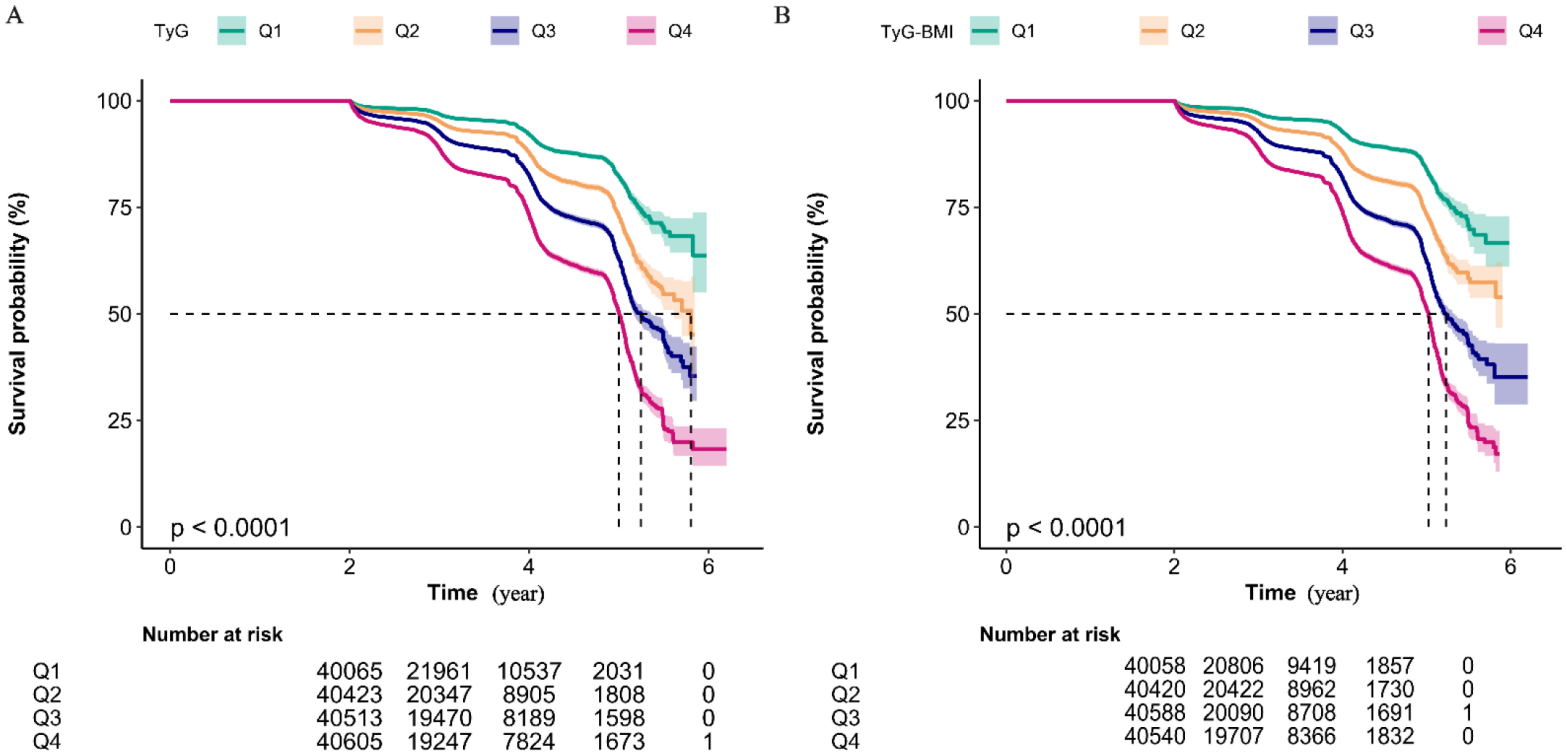
Kaplan-Meier Analysis of Cumulative Prediabetes Incidence by TyG Quartile Groups **(A)** and TyG-BMI Quartile Groups **(B)**. Kaplan-Meier curves showing the non-disease prevalence rate over time for four quartile groups (Q1 to Q4). The number of individuals at risk for each quartile group at non-disease status is indicated at specific time points (2, 4, 6 years). The log-rank test for intergroup comparison both yields the P-value of less than 0.01, suggesting a statistically significant difference in non-disease prevalence among the quartile groups. Notably, both the highest TyG group (Q4) and the highest TyG-BMI group (Q4) exhibits the highest cumulative prediabetes prevalence rate.

**Figure 4** displays Kaplan–Meier curves for the full cohort, illustrating cumulative incidence of prediabetes. For both TyG (**Figure 4A**) and TyG-BMI (**Figure 4B**), participants in the highest quartile (Q4) experienced a significantly higher cumulative incidence compared with all lower quartiles (log-rank *P* < 0.001 for each).

### Sensitivity analysis

Multivariable Cox regression analyses with multiple imputation for missing covariates yielded consistent results (**Supplementary Table 4**). Each 1-unit increase in the TyG index was associated with a 68% increased risk of developing prediabetes (HR, 1.68; 95% CI, 1.64–1.72; *P* < 0.001). The conclusions remained robust after excluding participants with a family history of diabetes (**Supplementary Table 5**) or baseline blood pressure abnormalities (**Supplementary Table 6**). Further analyses using TyG quintiles demonstrated unchanged associations (**Supplementary Table 7**). Finally, after removing extreme values (0.5th and 99.5th percentiles of TyG and TyG-BMI distributions), adjusted models showed essentially consistent results (**Supplementary Table 8**).

## Discussion

In this large, population-based longitudinal cohort of Chinese adults, we report several novel findings. First, both the TyG index and TyG-BMI showed an independent, positive association with incident prediabetes. Second, these exposures demonstrated nonlinear, dose-response relations with prediabetes risk and exhibited clear threshold effects; Kaplan–Meier curves confirmed that higher TyG and TyG-BMI levels were associated with greater cumulative incidence of prediabetes. Third, age and sex each modified these associations synergistically, such that the combination of younger age (< 45 years) or female sex with elevated TyG or TyG-BMI conferred a supra-additive increase in prediabetes risk. Finally, sensitivity analyses indicated that these associations remained robust under multiple alternative assumptions.

Previous studies have established the triglyceride–glucose (TyG) index as a reliable surrogate marker of IR, showing strong correlations with both the hyperinsulinemic–euglycemic clamp and the homeostasis model assessment of IR (HOMA-IR) (21, 22). Consequently, elevated TyG levels have been linked to increased risks of T2D and cardiovascular disease (23, 24). However, investigations specifically targeting prediabetes remain scarce and are often limited by small sample sizes or cross-sectional designs (25). The TyG-body mass index (TyG-BMI), which integrates adiposity with dysregulated lipid–glucose metabolism, has also emerged as a robust predictor of T2D and metabolic syndrome (14, 26), yet its association with incident prediabetes in large, ethnically diverse Chinese cohorts has not been adequately characterized.

The robust associations of the TyG index and TyG-BMI with incident prediabetes directly reflect their role as sensitive indicators of underlying IR. Elevated fasting TG signify increased free fatty acid flux and augmented hepatic very-low-density lipoprotein secretion, both key drivers of hepatic IR (27). Concomitant hyperglycemia denotes impaired insulin-mediated glucose disposal in skeletal muscle and adipose tissue (28). This lipid–glucose dysregulation establishes a vicious cycle: IR exacerbates dyslipidemia and hyperglycemia, which in turn further impair insulin signaling and heighten β-cell stress (29). By incorporating adiposity, TyG-BMI captures obesity’s contribution to IR—through pro-inflammatory adipokines (e.g., TNF-α, IL-6) and reduced adiponectin—amplifying systemic inflammation and metabolic dysfunction (30). Critically, IR with compensatory chronic hyperinsulinemia is not merely a precursor to dysglycemia, but an independent pathological state that precedes prediabetes/T2D by years or decades (31, 32). It directly drives systemic damage, including cardiovascular remodeling (e.g., atherosclerosis, hypertension), accelerated cellular senescence, oncogenesis, and neurodegeneration (33). Thus, the TyG index and TyG-BMI parsimoniously encapsulate IR-driven derangements in lipid-glucose-adipose axis that initiate both metabolic dysregulation and extra-glycemic organ damage, long before overt dysglycemia develops.

Strengths of this study are fourfold. First, TyG and TyG-BMI can be derived rapidly from routine fasting lipid and anthropometric data, permitting low-cost prediabetes risk stratification without additional testing. Second, the cohort spans multiple regions of China, is large, and covers a wide age range, enhancing generalizability to the broader Chinese population. Third, we evaluated additive interaction within age- and sex-specific strata, aligning with contemporary public health decision-making frameworks (34). Finally, extensive sensitivity analyses confirmed the stability of our findings. Collectively, these results provide concise, efficient tools for early identification of IR—a fundamental driver of cardiometabolic and multiorgan pathology. Their use in risk stratification extends beyond predicting dysglycemia; it flags a high-risk metabolic state where interventions (e.g., lifestyle, metformin) can potentially prevent not only diabetes but also IR-related cardiovascular, neoplastic, and neurological complications.

Several caveats merit consideration. First, although the cohort was not designed as a nationally representative sample, its multicenter design and broad age span enhance reasonable generalizability to the Chinese population. Second, the applicability of our findings to populations with substantially higher obesity prevalence (e.g., European or North American cohorts) requires verification. In such settings, the strong correlation between adiposity and dysglycemia may diminish the incremental predictive value of TyG over BMI or waist circumference alone, as adiposity may dominate the dysglycemia risk signal. However, TyG-BMI—by integrating metabolic dysregulation and adiposity—likely retains discriminative superiority for prediabetes risk stratification even in high-adiposity populations. This is mechanistically plausible as the combined index captures both lipid-glucose dysregulation and adiposity burden, thereby mitigating signal saturation effects from either pathway alone. Therefore, future multi-ethnic studies with stratified obesity profiles (e.g., ≥30% obesity) are warranted to establish population-specific thresholds. Moreover, in this study, prediabetes was defined solely on the basis of FPG; consequently, some incident cases may have been missed. Nevertheless, we observed a significant association of both TyG and TyG-BMI with prediabetes in this restricted sample. In addition, we acknowledge that the absence of glycated hemoglobin and oral glucose tolerance test data limits the generalizability and completeness of our findings. Future work should incorporate these additional metrics to provide a more comprehensive and accurate assessment of diabetes risk. Furthermore, residual confounding—stemming from imprecisely measured or unmeasured factors such as physical activity and diet—may persist despite extensive adjustment. Prediabetes was defined exclusively by incident impaired fasting glucose, potentially overlooking cases identified through alternative glycemic criteria. The relatively brief follow-up yielded a modest absolute event count; nevertheless, TyG and TyG-BMI retained significant and consistent associations with prediabetes, suggesting that their predictive utility is likely to endure—or strengthen—over longer observation periods. Despite these limitations, our findings underscore that TyG and TyG-BMI are more than prediabetes predictors—they are accessible proxies for IR, a root cause of systemic disease burden. Prioritizing IR detection—years before dysglycemia emerges—could transform early prevention paradigms by targeting the root cause of multiorgan damage.

## Conclusion

In this large, multicenter Chinese cohort, both the TyG index and TyG-BMI independently and non-linearly predicted incident prediabetes, with clear threshold effects at TyG of 9 and TyG-BMI of 200. The associations were amplified by older age and male sex, underscoring the value of sex- and age-specific risk stratification. Critically, as direct proxies for IR—an independent pathological state preceding dysglycemia by years—these indices empower clinicians to identify high-risk metabolic phenotypes for early interventions. Such interventions (ranging from intensive lifestyle optimization to targeted pharmacotherapy for eligible individuals) aim not only to prevent diabetes, but more importantly to mitigate IR-driven cardiovascular and neoplastic sequelae arising from chronic hyperinsulinemia.

## Data Availability

Publicly available datasets were analyzed in this study. This data can be found here: https://datadryad.org/dataset/doi:10.5061/dryad.ft8750v#citations.
