# Supplementary material for: Association between triglyceride-glucose and triglyceride glucose body mass index with risk of prediabetes: a multicenter Chinese medical examination cohort study
Source: Front Endocrinol (Lausanne). 2025 Oct 10;16:1668021. doi: 10.3389/fendo.2025.1668021 (PMC12549309; doi:10.3389/fendo.2025.1668021)
Supplement: Supplementary file 1 [file Table1.docx]

Supplemental Table 1 Sample Missing Data Overview

| Variable | Samples | Missing Samples | Missing Ratios, % |
| --- | --- | --- | --- |
| **Demographic** |  |  |  |
| Age | 179177 | 0 | 0.0000 |
| Sex | 179177 | 0 | 0.0000 |
| Family history of diabetes | 179177 | 0 | 0.0000 |
| **Health Status** |  |  |  |
| SBP | 179161 | 16 | 0.0089 |
| DBP | 179160 | 17 | 0.0095 |
| **Anthropometric Measures** |  |  |  |
| BMI | 179177 | 0 | 0.0000 |
| FPG | 179177 | 0 | 0.0000 |
| TC | 179176 | 1 | 0.0006 |
| TG | 179177 | 0 | 0.0000 |
| ALT | 177765 | 1412 | 0.7880 |
| BUN | 163172 | 16005 | 8.9325 |
| Cr | 170984 | 8193 | 4.5726 |
| TyG | 179177 | 0 | 0.0000 |
| TyG-BMI | 179177 | 0 | 0.0000 |

SBP: systolic blood pressure, DBP: diastolic blood pressure, BMI: body mass index, FPG: fasting plasma glucose, TC: total cholesterol, TG: triglyceride, ALT: alanine aminotransferase, BUN: blood urea nitrogen, Cr: creatinine, TyG: triglyceride-glucose index, TyG-BMI: TyG with body mass index

Supplemental Table 2 Association of covariates and prediabetes

| Variable | HR (95%CI) | *P* value |
| --- | --- | --- |
| Age, y | 1.03 (1.03~1.03) | < 0.001 |
| Sex |  |  |
| Male | Reference |  |
| Female | 0.64 (0.62~0.66) | < 0.001 |
| SBP, mmHg | 1.02 (1.02~1.03) | < 0.001 |
| DBP, mmHg | 1.03 (1.03~1.03) | < 0.001 |
| ALT, U/L | 1.0034 (1.0031~1.0036) | < 0.001 |
| BUN, mmol/L | 1.14 (1.13~1.15) | < 0.001 |
| Cr, umol/L | 1.0061 (1.0058~1.0065) | < 0.001 |
| Family history of diabetes |  |  |
| No | Reference |  |
| Yes | 1.06 (0.96~1.16) | 0.243 |
| TyG | 2.12 (2.08~2.17) | < 0.001 |
| TYG-BMI | 1.01 (1.01~1.01) | < 0.001 |

Supplemental Table 3 Multiplicative and additive interactions of TyG Index and TyG-BMI Index with Age and Sex on Prediabetes Risk

| Groups | HR (95% CI) | Multiplicative interaction HR (95% CI) | RERI (95% CI) | AP (95% CI) | SI (95% CI) |
| --- | --- | --- | --- | --- | --- |
| **TyG+Age^a^** |  | 0.80 (0.75~0.85) | 0.14 (0.02~0.26) | 0.05 (0.01~0.09) | 1.07 (1.01~1.14) |
| TyG (<8.27)+Age (<45 y) | Ref |  |  |  |  |
| TyG (<8.27)+Age (≥45 y) | 1.93 (1.83~2.04) |  |  |  |  |
| TyG (≥8.27)+Age (<45 y) | 1.95 (1.87~2.04) |  |  |  |  |
| TyG (≥8.27)+Age (≥45 y) | 3.02 (2.89~3.15) |  |  |  |  |
| **TyG+Sex^b^** |  | 1.21 (1.13~1.29) | 0.14 (0.06~0.21) | 0.09 (0.04~0.14) | 1.35 (1.12~1.64) |
| TyG (<8.27)+Sex (Male) | Ref |  |  |  |  |
| TyG (<8.27)+Sex (Female) | 0.79 (0.75~0.83) |  |  |  |  |
| TyG (≥8.27)+ Sex (Male) | 1.60 (1.53~1.66) |  |  |  |  |
| TyG (≥8.27)+ Sex (Female) | 1.52 (1.44~1.60) |  |  |  |  |
| **TyG-BMI+Age^a^** |  | 0.82 (0.77~0.88) | 0.18 (0.06~0.30) | 0.06 (0.02~0.10) | 1.10 (1.03~1.17) |
| TyG (<188.12)+Age (<45 y) | Ref |  |  |  |  |
| TyG (<188.12)+Age (≥45 y) | 1.92 (1.81~2.02) |  |  |  |  |
| TyG (≥188.12)+Age (<45 y) | 1.91 (1.82~2.00) |  |  |  |  |
| TyG (≥188.12)+Age (≥45 y) | 3.00 (2.87~3.14) |  |  |  |  |
| **TyG-BMI+Sex^b^** |  | 1.38 (1.29~1.47) | 0.28 (0.21~0.35) | 0.19 (0.14~0.23) | 2.21 (1.60~3.05) |
| TyG-BMI (<188.12) + Sex (Male) | Ref |  |  |  |  |
| TyG-BMI (<188.12)+ Sex (Female) | 0.73 (0.69~0.78) |  |  |  |  |
| TyG-BMI (≥188.12)+ Sex (Male) | 1.50 (1.43~1.57) |  |  |  |  |
| TyG-BMI (≥188.12)+ Sex (Female) | 1.52 (1.44~1.60) |  |  |  |  |

TyG: triglyceride-glucose index, TyG-BMI: TyG with body mass index, RERI: relative excess risk due to interaction, AP: attributable proportion, SI: synergy index

^a^ Adjusted for sex, family history of diabetes, systolic blood pressure, diastolic blood pressure, alanine aminotransferase, blood urea nitrogen, creatinine

^b^ Adjusted for age, family history of diabetes, systolic blood pressure, diastolic blood pressure, alanine aminotransferase, blood urea nitrogen, creatinine

Supplemental Table 4 Association of TyG and TyG-BMI with the risk of prediabetes (Multiple imputation)

| Categories | Model 1 | | Model 2 | | Model 3 | | Model 4 | |
| --- | --- | --- | --- | --- | --- | --- | --- | --- |
|  | HR (95% CI) | *P* value | HR (95% CI) | *P* value | HR (95% CI) | *P* value | HR (95% CI) | *P* value |
| TyG | 2.12 (2.08~2.17) | <0.001 | 1.80 (1.76~1.85) | <0.001 | 1.70 (1.66~1.74) | <0.001 | 1.68 (1.64~1.72) | <0.001 |
| TyG quartile |  |  |  |  |  |  |  |  |
| Q1 | 1(Ref) |  | 1(Ref) |  |  |  |  |  |
| Q2 | 1.58 (1.50~1.66) | <0.001 | 1.4 (1.33~1.47) | <0.001 | 1.36 (1.29~1.43) | <0.001 | 1.36 (1.29~1.42) | <0.001 |
| Q3 | 2.37 (2.26~2.48) | <0.001 | 1.89 (1.81~1.99) | <0.001 | 1.79 (1.71~1.88) | <0.001 | 1.78 (1.69~1.86) | <0.001 |
| Q4 | 3.67 (3.51~3.83) | <0.001 | 2.68 (2.56~2.81) | <0.001 | 2.43 (2.32~2.55) | <0.001 | 2.38 (2.27~2.50) | <0.001 |
| *P* for trend |  | <0.001 |  | <0.001 |  | <0.001 |  | <0.001 |
| TyG-BMI | 1.01 (1.01~1.01) | <0.001 | 1.01 (1.01~1.01) | <0.001 | 1.01 (1.01~1.01) | <0.001 | 1.01 (1.01~1.01) | <0.001 |
| TyG-BMI quartile |  |  |  |  |  |  |  |  |
| Q1 | 1(Ref) |  | 1(Ref) |  |  |  |  |  |
| Q2 | 1.68 (1.60~1.77) | <0.001 | 1.46 (1.38~1.54) | <0.001 | 1.42 (1.34~1.49) | <0.001 | 1.41 (1.33~1.48) | <0.001 |
| Q3 | 2.65 (2.52~2.78) | <0.001 | 2.04 (1.94~2.15) | <0.001 | 1.9 (1.81~2.00) | <0.001 | 1.88 (1.78~1.97) | <0.001 |
| Q4 | 3.95 (3.77~4.14) | <0.001 | 2.89 (2.75~3.03) | <0.001 | 2.54 (2.41~2.67) | <0.001 | 2.46 (2.34~2.59) | <0.001 |
| *P* for trend |  | <0.001 |  | <0.001 |  | <0.001 |  | <0.001 |

TyG: triglyceride-glucose index, TyG-BMI: TyG with body mass index, HR: hazard ratio, CI: confidence interval

Model 1: not adjusted

Model 2: adjusted for age, sex, family history of diabetes

Model 3: adjusted for model 2, additionally adjusted for systolic blood pressure, diastolic blood pressure,

Model 4: adjusted for model 3, additionally adjusted for alanine aminotransferase, blood urea nitrogen, creatinine

Supplemental Table 5 Association of TyG and TyG-BMI with the risk of prediabetes (excluding participants with a family history of diabetes, *N*=158344)

| Categories | Model 1 | | Model 2 | | Model 3 | | Model 4 | |
| --- | --- | --- | --- | --- | --- | --- | --- | --- |
|  | HR (95% CI) | *P* value | HR (95% CI) | *P* value | HR (95% CI) | *P* value | HR (95% CI) | *P* value |
| TyG | 2.13 (2.08~2.18) | <0.001 | 1.80 (1.76~1.85) | <0.001 | 1.70 (1.66~1.74) | <0.001 | 1.68 (1.63~1.72) |  |
| TyG quartile |  |  |  |  |  |  |  |  |
| Q1 | 1(Ref) |  | 1(Ref) |  | 1(Ref) |  | 1(Ref) |  |
| Q2 | 1.59 (1.51~1.68) | <0.001 | 1.40 (1.33~1.48) | <0.001 | 1.36 (1.29~1.44) | <0.001 | 1.36 (1.29~1.43) | <0.001 |
| Q3 | 2.39 (2.27~2.51) | <0.001 | 1.90 (1.80~2.00) | <0.001 | 1.80 (1.71~1.89) | <0.001 | 1.78 (1.69~1.87) | <0.001 |
| Q4 | 3.70 (3.53~3.88) | <0.001 | 2.69 (2.56~2.83) | <0.001 | 2.44 (2.32~2.57) | <0.001 | 2.39 (2.27~2.51) | <0.001 |
| *P* for trend |  | <0.001 |  | <0.001 |  | <0.001 |  | <0.001 |
| TyG-BMI | 1.01 (1.01~1.01) | <0.001 | 1.01 (1.01~1.01) | <0.001 | 1.01 (1.01~1.01) | <0.001 | 1.01 (1.01~1.01) |  |
| TyG-BMI quartile |  |  |  |  |  |  |  |  |
| Q1 | 1(Ref) |  | 1(Ref) |  | 1(Ref) |  | 1(Ref) |  |
| Q2 | 1.70 (1.61~1.80) | <0.001 | 1.47 (1.39~1.55) | <0.001 | 1.42 (1.35~1.51) | <0.001 | 1.41 (1.34~1.49) | <0.001 |
| Q3 | 2.66 (2.53~2.80) | <0.001 | 2.04 (1.93~2.16) | <0.001 | 1.90 (1.80~2.01) | <0.001 | 1.87 (1.77~1.98) | <0.001 |
| Q4 | 3.98 (3.79~4.19) | <0.001 | 2.89 (2.74~3.05) | <0.001 | 2.55 (2.41~2.69) | <0.001 | 2.46 (2.33~2.60) | <0.001 |
| *P* for trend |  | <0.001 |  | <0.001 |  | <0.001 |  | <0.001 |

TyG: triglyceride-glucose index, TyG-BMI: TyG with body mass index, HR: hazard ratio, CI: confidence interval

Model 1: not adjusted

Model 2: adjusted for age, sex

Model 3: adjusted for model 2, additionally adjusted for systolic blood pressure, diastolic blood pressure,

Model 4: adjusted for model 3, additionally adjusted for alanine aminotransferase, blood urea nitrogen, creatinine

Supplemental Table 6 Association of TyG and TyG-BMI with the risk of prediabetes (excluding participants whose SBP ≥ 140 mmHg or DBP ≥ 90 mmHg, *N* = 142190)

| Categories | Model 1 | | Model 2 | | Model 3 | |
| --- | --- | --- | --- | --- | --- | --- |
|  | HR (95% CI) | *P* value | HR (95% CI) | *P* value | HR (95% CI) | *P* value |
| TyG | 2.13 (2.08~2.19) | <0.001 | 1.82 (1.77~1.87) | <0.001 | 1.80 (1.75~1.85) |  |
| TyG quartile |  |  |  |  |  |  |
| Q1 | 1(Ref) |  | 1(Ref) |  | 1(Ref) |  |
| Q2 | 1.56 (1.47~1.65) | <0.001 | 1.40 (1.32~1.48) | <0.001 | 1.40 (1.32~1.48) | <0.001 |
| Q3 | 2.31 (2.19~2.44) | <0.001 | 1.89 (1.79~1.99) | <0.001 | 1.88 (1.77~1.98) | <0.001 |
| Q4 | 3.56 (3.38~3.74) | <0.001 | 2.67 (2.53~2.81) | <0.001 | 2.62 (2.48~2.76) | <0.001 |
| *P* for trend |  | <0.001 |  | <0.001 |  | <0.001 |
| TyG-BMI | 1.01 (1.01~1.01) | <0.001 | 1.01 (1.01~1.01) | <0.001 | 1.01 (1.01~1.01) |  |
| TyG-BMI quartile |  |  |  |  |  |  |
| Q1 | 1(Ref) |  | 1(Ref) |  | 1(Ref) |  |
| Q2 | 1.68 (1.59~1.79) | <0.001 | 1.47 (1.39~1.56) | <0.001 | 1.47 (1.38~1.55) | <0.001 |
| Q3 | 2.63 (2.49~2.78) | <0.001 | 2.07 (1.96~2.19) | <0.001 | 2.04 (1.93~2.17) | <0.001 |
| Q4 | 3.80 (3.60~4.00) | <0.001 | 2.86 (2.70~3.03) | <0.001 | 2.77 (2.62~2.94) | <0.001 |
| *P* for trend |  | <0.001 |  | <0.001 |  | <0.001 |

TyG: triglyceride-glucose index, TyG-BMI: TyG with body mass index, HR: hazard ratio, CI: confidence interval

Model 1: not adjusted

Model 2: adjusted for age, sex, family history of diabetes

Model 3: adjusted for model 2, additionally adjusted for alanine aminotransferase, blood urea nitrogen, creatinine

Supplemental Table 7 Association of TyG and TyG-BMI with the risk of prediabetes (TyG Quintile and TyG-BMI Quintile)

| Categories | Model 1 | | Model 2 | | Model 3 | | Model 4 | |
| --- | --- | --- | --- | --- | --- | --- | --- | --- |
|  | HR (95% CI) | *P* value | HR (95% CI) | *P* value | HR (95% CI) | *P* value | HR (95% CI) | *P* value |
| TyG Quintile |  |  |  |  |  |  |  |  |
| Q1 | 1(Ref) |  | 1(Ref) |  | 1(Ref) |  | 1(Ref) |  |
| Q2 | 1.52 (1.43~1.62) | <0.001 | 1.38 (1.30~1.46) | <0.001 | 1.35 (1.27~1.43) | <0.001 | 1.34 (1.27~1.43) | <0.001 |
| Q3 | 2.05 (1.94~2.17) | <0.001 | 1.70 (1.61~1.80) | <0.001 | 1.63 (1.54~1.73) | <0.001 | 1.62 (1.53~1.72) | <0.001 |
| Q4 | 2.88 (2.73~3.04) | <0.001 | 2.21 (2.08~2.33) | <0.001 | 2.07 (1.95~2.19) | <0.001 | 2.04 (1.93~2.16) | <0.001 |
| Q5 | 4.10 (3.89~4.32) | <0.001 | 2.94 (2.78~3.11) | <0.001 | 2.65 (2.51~2.80) | <0.001 | 2.59 (2.45~2.74) | <0.001 |
| *P* for trend |  | <0.001 |  | <0.001 |  | <0.001 |  | <0.001 |
| TyG-BMI Quintile |  |  |  |  |  |  |  |  |
| Q1 | 1(Ref) |  | 1(Ref) |  | 1(Ref) |  | 1(Ref) |  |
| Q2 | 1.58 (1.49~1.69) | <0.001 | 1.41 (1.32~1.50) | <0.001 | 1.38 (1.29~1.47) | <0.001 | 1.37 (1.29~1.47) | <0.001 |
| Q3 | 2.35 (2.21~2.49) | <0.001 | 1.88 (1.77~2.00) | <0.001 | 1.79 (1.68~1.90) | <0.001 | 1.77 (1.66~1.88) | <0.001 |
| Q4 | 3.21 (3.03~3.40) | <0.001 | 2.38 (2.24~2.52) | <0.001 | 2.19 (2.06~2.33) | <0.001 | 2.15 (2.02~2.29) | <0.001 |
| Q5 | 4.53 (4.28~4.79) | <0.001 | 3.26 (3.08~3.46) | <0.001 | 2.86 (2.69~3.04) | <0.001 | 2.76 (2.60~2.94) | <0.001 |
| *P* for trend |  | <0.001 |  | <0.001 |  | <0.001 |  | <0.001 |

TyG: triglyceride-glucose index, TyG-BMI: TyG with body mass index, HR: hazard ratio, CI: confidence interval

Model 1: not adjusted

Model 2: adjusted for age, sex, family history of diabetes

Model 3: adjusted for model 2, additionally adjusted for systolic blood pressure, diastolic blood pressure,

Model 4: adjusted for model 3, additionally adjusted for alanine aminotransferase, blood urea nitrogen, creatinine

Supplemental Table 8 Association of TyG and TyG-BMI with the risk of prediabetes ((Minorized distributions of TyG and TyG-BMI at the 0.5 and 99.5 percentiles, *N*=158616)

| Categories | Model 1 | | Model 2 | | Model 3 | | Model 4 | |
| --- | --- | --- | --- | --- | --- | --- | --- | --- |
|  | HR (95% CI) | *P* value | HR (95% CI) | *P* value | HR (95% CI) | *P* value | HR (95% CI) | *P* value |
| TyG | 2.24 (2.18~2.29) | <0.001 | 1.86 (1.81~1.92) | <0.001 | 1.76 (1.71~1.81) | <0.001 | 1.74 (1.69~1.79) | <0.001 |
| TyG quartile |  |  |  |  |  |  |  |  |
| Q1 | 1(Ref) |  | 1(Ref) |  | 1(Ref) |  | 1(Ref) |  |
| Q2 | 1.57 (1.49~1.66) | <0.001 | 1.39 (1.32~1.47) | <0.001 | 1.36 (1.29~1.43) | <0.001 | 1.35 (1.28~1.43) | <0.001 |
| Q3 | 2.31 (2.20~2.43) | <0.001 | 1.85 (1.76~1.95) | <0.001 | 1.76 (1.67~1.85) | <0.001 | 1.74 (1.66~1.84) | <0.001 |
| Q4 | 3.56 (3.40~3.72) | <0.001 | 2.60 (2.48~2.73) | <0.001 | 2.38 (2.26~2.50) | <0.001 | 2.33 (2.22~2.45) | <0.001 |
| *P* for trend |  | <0.001 |  | <0.001 |  | <0.001 | 1(Ref) | <0.001 |
| TyG-BMI | 1.01 (1.01~1.01) | <0.001 | 1.01 (1.01~1.01) | <0.001 | 1.01 (1.01~1.01) | <0.001 | 1.01 (1.01~1.01) |  |
| TyG-BMI quartile |  |  |  |  |  |  |  |  |
| Q1 | 1(Ref) |  | 1(Ref) |  | 1(Ref) | <0.001 | 1(Ref) |  |
| Q2 | 1.65 (1.56~1.75) | <0.001 | 1.43 (1.35~1.51) | <0.001 | 1.39 (1.31~1.47) | <0.001 | 1.38 (1.30~1.46) | <0.001 |
| Q3 | 2.57 (2.45~2.71) | <0.001 | 1.99 (1.89~2.10) | <0.001 | 1.86 (1.76~1.96) | <0.001 | 1.83 (1.73~1.93) | <0.001 |
| Q4 | 3.78 (3.60~3.96) | <0.001 | 2.76 (2.62~2.90) | <0.001 | 2.44 (2.31~2.57) | <0.001 | 2.36 (2.24~2.50) | <0.001 |
| *P* for trend |  | <0.001 |  | <0.001 |  | <0.001 |  | <0.001 |

TyG: triglyceride-glucose index, TyG-BMI: TyG with body mass index, HR: hazard ratio, CI: confidence interval

Model 1: not adjusted

Model 2: adjusted for age, sex, family history of diabetes

Model 3: adjusted for model 2, additionally adjusted for systolic blood pressure, diastolic blood pressure,

Model 4: adjusted for model 3, additionally adjusted for alanine aminotransferase, blood urea nitrogen, creatinine
